# Analysis of Primary Stability of Dental Implants Inserted in Different Substrates Using the Pullout Test and Insertion Torque

**DOI:** 10.1155/2013/194987

**Published:** 2013-01-22

**Authors:** Nathalia Ferraz Oliscovicz, Antônio Carlos Shimano, Élcio Marcantonio Junior, César Penazzo Lepri, Andréa Candido dos Reis

**Affiliations:** ^1^Department of Dental Materiais and Prosthodontics, University of São Paulo, Av. do Café s/n, 14040-904 Ribeirão Preto, SP, Brazil; ^2^Department of Biomechanics, Medicine and Rehabilitation of the Locomotor, University of São Paulo, Av. Bandeirantes, 3900, 14049-900 Ribeirão Preto, SP, Brazil; ^3^Department of Diagnostic and Surgery, UNESP, R. Humaitá, 1680, 14801-903 Araraquara, SP, Brazil; ^4^Department of Restorative Dentistry, University of São Paulo, Av. do Café s/n, 14040-904 Ribeirão Preto, SP, Brazil

## Abstract

The aim of the study was to evaluate mechanical behavior of implants inserted in three substrates, by measuring the pullout strength and the relative stiffness. 32 implants (Master Porous-Conexao, cylindrical, external hexagon, and surface treatment) were divided into 4 groups (*n* = 8): pig rib bone, polyurethane Synbone, polyurethane Nacional 40 PCF, and pinus wood. Implants were installed with the exact distance of 5 mm of another implant. The insertion torque (N*·*cm) was quantified using the digital Kratos torque meter and the pullout test (N) was performed by an axial traction force toward the long axis of the implant (2 min/mm) through mount implant devices attached to a piece adapted to a load cell of 200 Kg of a universal testing machine (Emic DL10000). Data of insertion torque and maximum pullout force were submitted to one-way ANOVA and Bonferroni tests (*α* = 0.05). Polyurethane Nacional 40 PCF and pinus wood showed the highest values of insertion torque and pullout force, with significant statistical difference (*P* < 0.05) with other groups. The analysis showed stiffness materials with the highest values for primary stability.

## 1. Introduction 

Over the last few years, dental implants have shown satisfactory success rates in several clinical situations [1]. For favorable prognosis, some requirements are needed such as the absence of mobility of the implant during surgical procedure, which is defined as primary stability. This property is essential for the maintenance of the peri-implant hard and soft tissues [2–4] and, for this reason, it is a determining factor for complete osseointegration [5–7].

Good primary stability allows balanced distribution of masticatory effort and functional occlusal loads [8] soon after the implant is inserted. This stability may decrease over time due to peri-implant bone remodeling [9]. For long-term success in Implant Dentistry, the following factors related to the patient must be taken into consideration: bone quality and volume, peri-implant clinical parameters, implant stability [6, 9], factors related to the surgical technique, and the selection of implants. These previously assessed factors provide predictable osseointegration and if load can be applied or not.

Some methods for assessing primary stability have already been proposed, as insertion torque and resonance frequency, but no methods show completely precise results. Medical orthopedics proposes a method to simulate this analysis, the pullout test, which despite the need for specific devices for each type of implant, it is established in this medical specialty [10–17]. Moreover, it is necessary to select materials to be used as a substrate for these tests in view of the disadvantages found in selecting similar substrates to the human bone [1, 18, 19] as well as the difficult access to good *in vivo *quality bone, which is a prerequisite for the initial fixation [20]. 

The variety of cadaver bones and variables that influence quality, such as fenestrations, which are frequent and unknown in these bones, may influence reliability and validity of the measurements [19], making it difficult to conduct the study due to the need for large samples to obtain significant results [1]. Furthermore, the use of human bones presents difficulties with regard to availability, preparation, and preservation. These conditions have led science to seek for substitutes for the human bone, which led to the development of synthetic bones that allow the standardization of mechanical testing and increased availability. The use of polyurethane models began with Uta for analysis of the femur [19]. Over the years, anisotropy and heterogeneity, which are characteristics belonging to the human bone, have improved and composite models [19, 20] have been proposed. At present, the most complete validation of these models has been described by Cristofolini et al.  [18],  who  compared  synthetic  bone with the human bone, emphasizing the mechanical properties such as viscoelastic behavior, main deflection and strain distribution under axial loading, bending, and torsion for the orthopedics area.

In addition to the polyurethane, wood *Araucaria augustifolia*, basic density from 0.42 to 0.48 g/cm³ [21], similar to the averages of the bones of the mandible and maxilla [22], is also suggested as a material for the analysis of implant fixation to be anisotropic [23] and resembles human bone sensation perceived in the drilling and installation [24]. 

Based on these concepts, the aim of this study was to propose the evaluation of the mechanical behavior of dental implants inserted in different substrates, in order to determine an experimental model of dental implants using pullout tests. 

## 2. Material and Methods 

Four types of materials used as substitutes for human bone were selected for this study. The samples were divided into three groups (*n* = 8) according to the type of substrate of the test specimen: pig rib bone, polyurethane Synbone (Synbone AG, Malans, Switzerland); polyurethane Nacional (Nacional Ossos, Jaú, Brazil); and wood of the species of *Araucaria angustifolia*. The cylindrical implants of the type Master Porous of the brand Conexão (Conexão Sistemas de Prótese, Arujá, Brazil) measuring 11.5 mm in height and 3.75 mm in diameter with an external hexagon and Porous surface treatment were used. 

The implants were inserted in pig rib bone segments with 20 mm long, approximately the same thickness of cortical and spongy bone, and included in PVC cylinder (20 mm diameter and 15 mm height), filled with acrylic resin, such that 5 mm of bone stay outside of the cylinder, not included by the material. Samples were kept under freezing (−21 ± 5°C) to maintain their physical properties and, previously to surgical procedures and measurements of implants, remained at ambient temperature for 2 hours to reach thermal equilibrium and not change biomechanical properties. Others implants were inserted in a rectangular polyurethane block Nacional with a density of 40 PCF and measuring 4.2 mm in height, 17.8 mm wide, and 13 mm in length. An artificial bone specimen similar to the human femur in shape and density of the brand Synbone was selected. Eight wood cylindrical test specimens measuring 30 mm in diameter and 13 mm in length were fabricated in the Precision Workshop of the School of Medicine of Ribeirão Preto in order to facilitate mechanical testing and prevent future damages to the screws and test specimens.

The test specimens of a specific group were perforated and the implants were inserted by a single operator with the aid of a counter-angle, a surgical electric motor MC 101 Omega adjusted to 45 N and 1861 rpm, both of the brand Dentscler (Dentscler Indústria de Aparelhos Odontológicos Ltda., Ribeirão Preto, Brazil), and a set of cutters and accessories from the Connect Master Kit (Conexão Sistemas de Prótese, Arujá, Brazil), following the sequence of steps recommended by the manufacturer. 

Each implant was inserted in the test specimens with the exact distance of 5 mm from the other implant in order to prevent possible alterations in the material after performing the test causing interference in the analysis of adjacent implant. Group 1 received the implants in the central portion of the block in order to avoid interference that the press on the sides of the material might cause to the results. Group 2 had the implants placed in the upper portion of the femoral head that had a strip of cortical bone with an average thickness of 2 mm and a medullary portion with an average thickness of 15 mm. In Group 3, the implants were inserted in the central part of the diameter of each cylindrical test specimen. After the implants were inserted and tested in the specific group, they were removed with the aid of the torque meter Connection applying the reverse torque and then the test specimens were inserted in the other group to test the pullout strength. 

**Figure 1:**
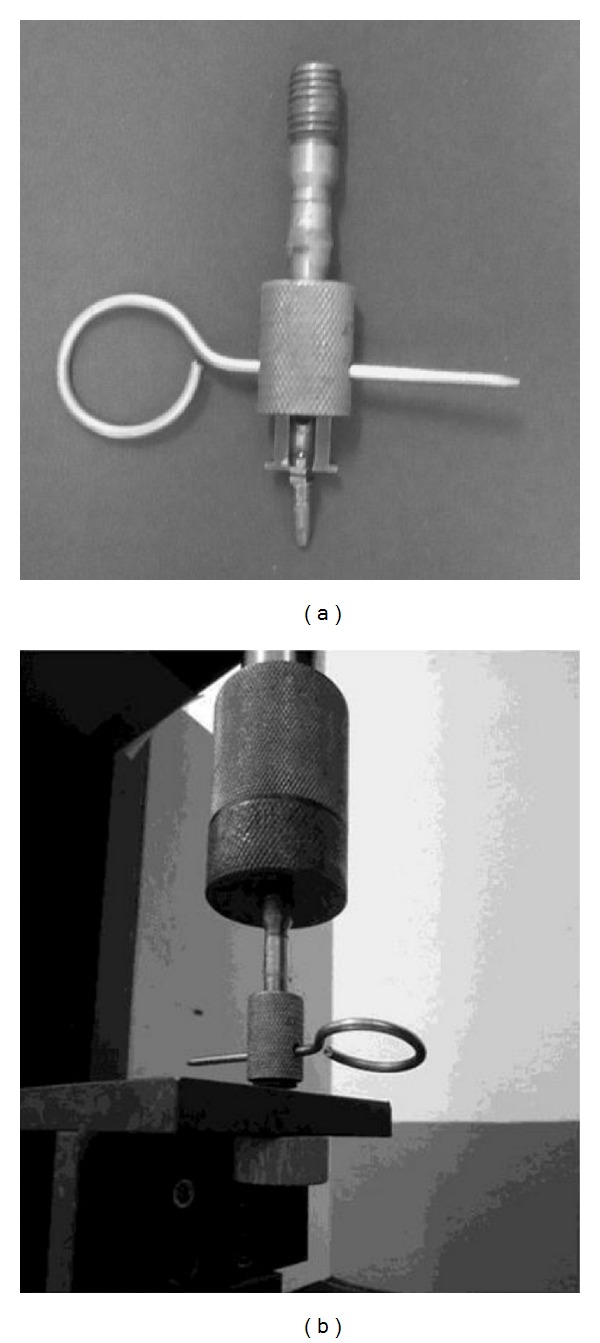
Accessories and devices for pullout test—axial traction force in the long axis of the implant.

Insertion torque analysis was made in digital torquimeter Kratos (Kratos Industrial Equipment Inc., Cotia, SP, Brazil) coupled to devices or implants mounts, adapted to the respective implants shapes (Figure 1(a)). Value was measured at each turn screw, being considered the maximum value obtained, which was converted to N·cm by the formula: *y* = 0,0449*x* − 0.7907, where *y* is the value N·cm, and *x* is the value read. 

To verify the pullout strength, the mount implants of the Porous Master implant of the brand Conexão were used, which were coupled to a piece adapted to a load cell of 200 kg of a universal testing machine EMIC brand model DL10000 (Emic Equipamentos e Sistemas de Ensaios LTDA, São José dos Pinhais, Brazil) (Figure 1(a)). The pullout test was performed using an axial traction force in the direction of the long axis of the implant for 2 min/mm, obtaining maximum pullout force and the relative rigidity of the material (Figures 1(a) and 1(b)). 

Data were analyzed with regard to their distribution and homogeneity, and since they were normal (Kolmogorov-Smirnov test) and homogeneous (Levene test), the one-way analysis of variance was used (one-way ANOVA: material). To differentiate the means, the Bonferroni test was used at a level of significance of 5%. 

## 3. Results 

**Figure 2:**
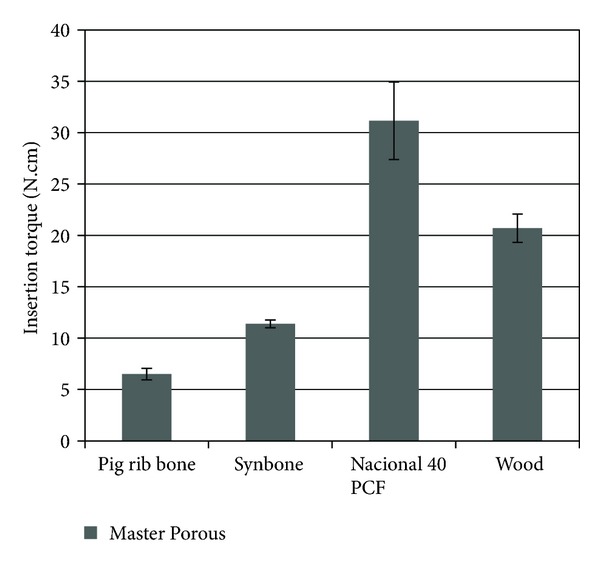
Statistical comparison of insertion torque among the four groups.

After statistical analysis of insertion torque, it was found that there was the highest average when the implant was inserted into the polyurethane National 40 PCF (31.15 ± 7.53) and in the wood (20.70 ± 2.77), with significant difference with all other substrates (*P* < 0.05) (Figure 2). 

**Figure 3:**
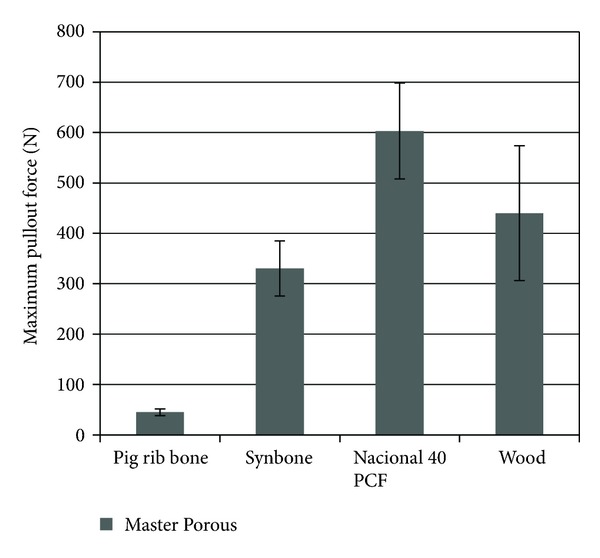
Statistical comparison of maximum pullout force among the four groups.

The results of maximum pullout force showed that there was a difference between the groups studied (*P* < 0.05). The implants inserted in the polyurethane National 40 PCF (603.10 ± 190.33 N) and wood (740.06 ± 268.13 N) showed higher pullout strength values with a statistically significant difference when compared with Synbone and natural bone (*P* < 0.05), as in insertion torque analysis. Implants inserted in bone possessed the lowest mean strength (44.74 ± 13.36 N) (Figure 3). 

## 4. Discussion 

The aim of most dental studies is to assess secondary stability or osseointegration. However, it is of great importance that more studies determine primary stability for it is an indicator of greater significance to guarantee of osseointegration [5, 6, 8]. 

The methods for measuring clinical primary stability, although feasible and useful, have limitations, and a method that correlates the different types of implants with its initial fixation is needed, similar to what occurs to the human bone with the use of a similar material for laboratory mechanical testing [18, 19]. 

The present study assessed four materials that could be indicated as substitutes for the human bone to perform the mechanical tests of dental implants with the purpose of developing a new experimental model for the analysis of primary stability. Due to the difficulty in obtaining of human bone models with a homogeneous sample, the American Society for Testing Materials [25] has shown that polyurethane blocks have mechanical properties that mimic the human bone and this is considered the standard material for mechanical testing with orthopedic implants [16–19]. Some authors have used polyurethane blocks for analyzing primary stability of dental implants [8, 22] using methods such as resonance frequency analysis and insertion torque. Thus, the selection of polyurethane materials used in this study derived from the analysis of similar studies [13, 16, 17] to assess the insertion torque and pullout or tensile strength of dental implants since they showed similar results when compared with the human cadaver bone [18, 19].

The choice of wood was due to its similar characteristics to the bone, which were perceived through tactile sensitivity during perforation of the substrate, and its intrinsic anisotropic properties, homogeneity and uniformity. This quality may explain the results presented in this study, which showed that wood presented high mean insertion torque and pullout strength when compared with the other materials, as Synbone and wood. 

Different methods for objectively assessing primary stability have been proposed. However, there is an absence of a gold standard for this analysis. Periotest (Siemens AG, Germany, Bensheim) is an instrument that has been proposed to measure primary stability, but it was not considered an ideal tool for this evaluation due to its inability to respond to minor changes at the bone-implant interface [26]. Several studies have reported that resonance frequency analysis is a useful tool to analyze primary stability after implantation, as well as the degree of stability after osseointegration. However, the interpretation of the values of the implant stability quotient (ISQ) still lacks scientific knowledge and there is no consensus of a presupposed value for high or sufficient primary stability for immediate loading. Furthermore, the ISQ values of different implant systems cannot be compared [27]. 

Another widely used method described by Friberg to measure primary stability [20] is insertion torque. Our study used this method as standard analysis of primary stability, that it can be used to assess more accurately bone quality and support, which are measured at the time of final seating of the implant in the receptor bed [6, 27]. Determining insertion torque is one of the most reliable [5] methods to obtain information about bone quality. Although insertion torque is proposed by many scholars, comparability among different implant systems is still unclear, and the minimum level of primary stability needed for immediate loading has not been defined [1, 27].

Thus, besides the insertion torque, widely used for analysis of primary stability of dental implants, the method pullout test was used, which is widely used in orthopedic implants for the analysis of mechanical resistance [10–17], using natural and synthetic bones or substrates that satisfy the reproduction of the human bone tissue to measure resistance of the screw [16]. Although the physiological cyclic forces on the implants in a mandible or maxilla are not only limited to the axial forces shown in the test, this method allows the parameters studied to be assessed through maximum pullout force as well as comparing the different implants and types of implantation [13]. 

Studies have reported that screws have the capacity to resist to pullout strength, mechanical property related to the geometry and size of the screw, bone quality and quantity [1, 13], insertion torque [10, 11], and pilot orifice preparation [11, 13, 28]. Furthermore, the surface of the screw in contact with the bone tissue and the number of threads per unit length of the screw is proportional [10]. 

All these variables suggest pullout strength as a predictor of primary stability. The correlation of stability with design and surface treatment of implants allows different screws to be idealized in order to optimize osseointegration, help indication of implants, and reduce the indication of bone grafts to facilitate the surgical technique, although little has been done to apply these assumptions in the development of new projects for screws. 

Despite the paucity of studies in the literature on the subject proposed, the experimental model showed that the pullout test can provide important data, such as initial stability through maximum pullout force, since the measurement method is still controversial [5, 13, 27]. It may also provide information about the substrate behavior obtained from the results such as relative rigidity and deformation. 

Thus, the pullout test associated with the use of a substrate that mimics the real conditions of bone enables conducting laboratory studies that correlate mechanical properties with the shape and surface treatment of the implant, allowing great industrial advance, since these results may lead to the proposal of new models of screws. 

The analysis of the mechanical behavior of different implants inserted in substrates of human bone substitutes showed the polyurethane Nacional 40 PCF and wood with the highest values for insertion torque and maximum pullout strength. Therefore, bone density influenced this analysis, seen by the highest torque and resistance pullout in materials compared to bone type D2 [8, 24], and the lowest values in bones less dense because they have a density similar to bones D4, based on some studies that show that bones type D2 and D3,  reveal  no statistical difference between implants analyzed [29, 30] only between bones D2 and D4, and D3 and D4. Furthermore, the low density of the rib bone is evidenced by the fact they cannot be compared to bone cortical bone, which influences  the  density  of  the  substrate  [22, 27].
